# Trust-Based Cooperative Social System Applied to a Carpooling Platform for Smartphones

**DOI:** 10.3390/s17020245

**Published:** 2017-01-27

**Authors:** Cándido Caballero-Gil, Pino Caballero-Gil, Jezabel Molina-Gil, Francisco Martín-Fernández, Vincenzo Loia

**Affiliations:** 1 Departament of Computer Engineering and Systems, University of La Laguna, 38271 Tenerife, Spain; pcaballe@ull.es (P.C.-G.); jmmolina@ull.es (J.M.-G.); 2 IBM Research, 38271 Tenerife, Spain; fmartinfdez@gmail.com; 3 Dipartimento di Scienze Aziendali—Management & Innovation Systems/DISA-MIS, University of Salerno, 84084 Fisciano, Italy; loia@unisa.it

**Keywords:** security, carpooling, trust, privacy, mobile application, social driving

## Abstract

One of the worst traffic problems today is the existence of huge traffic jams in almost any big city, produced by the large number of commuters using private cars. This problem has led to an increase in research on the optimization of vehicle occupancy in urban areas as this would help to solve the problem that most cars are occupied by single passengers. The solution of sharing the available seats in cars, known as carpooling, is already available in major cities around the world. However, carpooling is still not considered a safe and reliable solution for many users. With the widespread use of mobile technology and social networks, it is possible to create a trust-based platform to promote carpooling through a convenient, fast and secure system. The main objective of this work is the design and implementation of a carpool system that improves some important aspects of previous systems, focusing on trust between users, and on the security of the system. The proposed system guarantees user privacy and measures trust levels through a new reputation algorithm. In addition to this, the proposal has been developed as a mobile application for devices using the Android Open Source Project.

## 1. Introduction

The recent and rapid increase in the number of vehicles has led to growing pollution, especially in large cities. This is one of the main causes of global warming, which is why many governments have begun to take steps to try to reduce it. The most common measures are the promotion of public transport and the limitation in the use of vehicles in city centres through the prohibition of cars with a single occupant or with even or odd number plates depending on the day of the week, etc. However, it could be said that so far none of these solutions has proven to be the most convenient and/or affordable solution for everybody.

Another practical solution would consist of avoiding the low occupancy of most vehicles in cities, through car sharing, so that empty seats are used in most trips. This modality is known by the term carpooling and has been proposed as an effective way to reduce pollution and cut expenses. A related but different approach, known as carsharing, is based on collective fleets of cars that can be temporally rented by multiple users, but such a solution does not solve the aforementioned problem because it does not imply that users share the vehicle at the same moment. There is another term, ridesharing, which is generally used to refer to different solutions to share the use of a car with other people in order to travel to a particular destination. In addition to carsharing and carpooling (also known as real-time or instant or dynamic or ad hoc ridesharing), ridesharing also includes other versions known as slugging, lift sharing and covoiturage. This work does not address ridesharing solutions different from carpooling.

Both types of collaborative solutions have been increasingly used since the beginning of the economic crisis, thanks to technology 2.0. They are applicable in many different cases, but are especially useful in environments and situations like universities, holidays, long journeys and urban centres. This is because, in these cases, both owners and passengers of vehicles have the same motivations to consider the carpooling solution. Their main goal is usually to share the cost of fuel, but they might have other reasons, such as trying to avoid parking problems, wanting to talk, meet new people or make a contribution to environmental protection.

One of the main problems of carpooling is reliability, due to the fact that the service requires users to be confident that the driver will take them to their destination, and that drivers be sure that the accompanying people will behave properly during the trip. A practical improvement applicable to existing carpooling systems is described here, based on the use of the latest technological advances in smartphones and social networks to increase reliability. The proposed solution first allows the establishment of trust between drivers and passengers through reputation accounting, and, second, protects the privacy of all users. This is the main aim of this work, but there are others related to carpooling such as route optimization or enhancement of cooperation, which are not addressed here.

This paper is organized as follows. Section 2 mentions several related works, and Section 3 introduces the general design of the proposal, which is mainly based on the reputation algorithm sketched in Section 4. In Section 5, the developed Android application is described. Section 6 briefly analyses the security of the proposal. Finally, some conclusions and open problems can be found in Section 7.

## 2. Related Works

Carpooling has a long history as it had a boom in the United States during World War II, and later, in the mid-1970s, it was once again used as a solution during the oil and energy crises [1]. However, in those days, without the technology available today, carpooling had to face many difficult obstacles, such as the need to develop a network of users and find convenient forms of communication. Gradually, the means used to organize the trips were changing from the telephone to other more flexible technologies like Internet, email and smartphones. Nowadays, there are many different carpooling platforms and services, but, even today, they can be considered in their early stages because none of them has reached a large mass of users.

Several features of various existing carpooling systems are shown in Table 1, including the most relevant security-related ones. In particular, this comparative analysis includes as representative systems: Amovens [2], Blablacar [3], Compartir.org [4], and ZimRide [5].

For instance, BlaBlaCar [3] is the largest carpooling network in Europe. It is a service focused on long distance trips and uses social networks for registration and feedback and as an enabler of real connections between users. The biggest carpooling service in the United States is ZimRide [5], where payments are made through credit card account and PayPal. The main system to build trust in all of these platforms is based on user-given points. However, ignoring this security system is quite easy because users who get a negative score, can create a new profile with new credentials and no points.

Apart from the aforementioned practical platforms, which are already in operation, there are several papers that propose different solutions for carpooling. Some of them are centred on route optimization, and others focus on cooperation enforcement or passenger matching, and others are centred in multi-agent platforms. First, the work [6] shows an integrated system with an optimization module, which applies heuristic methods to solve the organization of the routing problem in carpooling services by using different technologies such as Web, Geographic Information System (GIS) and Short Message Service (SMS). Second, the paper [7] defines a push service to promote carpooling through instant processing. The paper [8] presents a carpooling architecture that uses a credit mechanism to encourage cooperation among users, by defining a business model where such a credit can be used in parking facilities and can be bought with real money. A more recent work is [9], where an algorithm to encourage carpooling is proposed based on assigning priority to users with positive feedback through a fuzzy logic scheme. Thirdly, an automatic service to match commuting trips is presented in [10]. In addition, passenger matching is the topic of the paper [11], where a system is proposed to help users in choosing a transport solution according to its ecological footprint, matching their needs, preferences, and actual location. Finally, another interesting proposal is [12], based on a multi-agent platform that focuses on security services that allow the mutual authentication of the users and of the application components with the system.

This work differs from all the aforementioned because its main goal is trust, obtained through a combination of reputation measurements and privacy protection that can be used in existing carpooling services. Additionally, the proposed system has been tested through the implementation of an open-source carpooling platform where any driver can insert the availability of empty seats on his/her car for a planned trip and potential passengers can search for trips and contact him/her.

## 3. Description of the Carpooling Proposal

Although the main objective of the proposed design is to increase trust in users, there are also other factors that have been taken into account, such as privacy protection, user-friendliness and usability. Thus, one of the main features of the proposal is that users who publish their trips have their privacy fully protected. Unlike other carpooling platforms, in the described system, no user is allowed to access data such as email, phone, full name or other data of another user, unless it is authenticated on the platform, and the algorithm for checking mutual trust returns a valid permission for it. In this case, the interested user will be able to see the relevant data. Otherwise, it can only submit a request to another user (driver) so that this can decide whether the applicant is reliable enough to have access to those data.

The proposal is based on trust relationships in such a way that those who want to use it first have to authenticate it in the platform through some social network such as Facebook, Twitter or Google+. In this way, the proposal checks the existence of some chain of trust between the applicant and other users, based on the so-called rule of six degrees of separation [13]. This rule is the theory that everyone is six or fewer steps away from each other person in the world, so that a chain of ’a friend of a friend’ statements can be made to connect any two people in up to six steps.

In addition, the reputation gained through the use of the application is an influential factor considered in the decision on whether to share a car with some person. To do this, at the end of every shared travel, the application asks both driver and passengers to score the other users in the trip. Such scores are used in future trips so that seats offered by drivers with good scores are more likely to be selected than others with lower scores. Furthermore, well-scored passengers are more likely to have access to details of other users when selecting a new trip.

**Figure 1:**
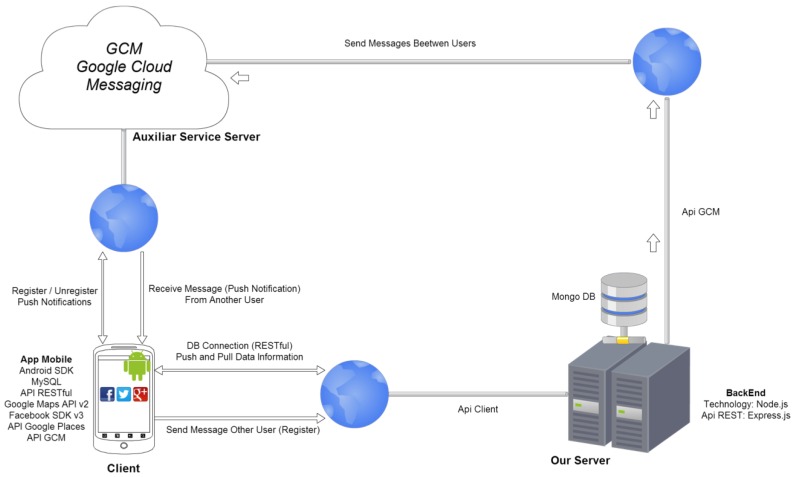
Architecture of the carpooling proposal.

The general architecture used in the proposed application model is known as client–server (see Figure 1). Its different elements are the following:

- Client: Mobile device used for the system.
- Server: Hosted in the cloud, and divided into two parts. The first part is the Google Cloud Messaging (GCM) server that handles all of the notifications and is responsible for sending the notification when the receiving clients are alive. The second part is the DataBase (DB) dedicated server, which stores all data related to users and the system in a database. It also serves as a gateway for sending notifications between the client and the GCM server.

The proposal protects user privacy through limited and controlled access to user data, according to the trust level stated for the relationship between each pair of users. This trust level is obtained through the combination of direct scores and information obtained from trust networks so that, by combining both sources, the system is provided with enough data to deduce whether people can trust each other or not. In this way, privacy is dealt with accordingly as one of the most important aspects of the proposed carpooling system.

The PageRank algorithm [14] used by Google Search to rank websites in their search engine results is used here as a first approach for the development of a trust measurement algorithm that provides a value to each pair of users. This algorithm is used here to predict whether two people can trust each other. However, since it does not fully fit the morphology of the specific problem, a second approach is also used here to complement it, based on Bayesian networks to know whether people can trust each other. The refined algorithm for trust measurement contained in the developed Android application is explained below.

## 4. The Reputation Algorithm

To the best of our knowledge, no existing carpooling proposal offers a quantitative method based on the theory of six degrees of separation to decide if two users should trust each other to share a car or not. Some of the proposals do not even allow drivers to decide who can or cannot apply their route. There are some proposals [15,16] that use a quantitative method based on the similarities among users. Their main problem is that they have to collect information about the attributes and characteristics of each user. The proposal aims to be simple for the user, so that he/she does not have to fill in any information about his/her attributes. With a simple click to enable social login on the network after a previous registration, the user can log into the system and start using the platform.

The reputation algorithm is the main feature of the proposal because, thanks to it, the user can use a quantitative measure to decide whether to trust another user or not. The algorithm is based on the theory of six degrees of separation and individual scores within our platform. This number of steps may be reduced significantly by introducing the concept of social networks. Our application uses social networks when logging into the application to create a network of user, which is used to interconnect users and provide a reliable measure of confidence among them. Through the use of social networks, the six degrees of separation can be reduced on average to less than four. In particular, according to several studies on Facebook [17,18,19], the average distance is 3.9, corresponding to intermediaries or ’degrees of separation’, which shows that the world is even smaller than expected.

The trust rate is a value between 0 and 1, shown to the user as a character (*A*, *B*, *C*, *E*, *F*) and computed for each pair of users to inform about the trust between them. This measure is calculated taking into account the two parameters explained below: degree of friendship and ratings of users in the platform.

The social network is shown as a directed weighted graph G=(V,E), where $V=\{v_{1},v_{2},...,v_{n}\}$ represents the set of users of the network, and $e_{ij}=v_{i}\to v_{j}\in E$ if $v_{i}$ is adjacent to $v_{j}$, which corresponds to $v_{i}$ being a friend of $v_{j}$.

Let $F=[df_{ij}]$ denote the weight matrix of *G* defined by Equation (1):

(1) $$df_{ij}=\left\{\begin{array}{cc} \frac{\sum (FA_{ij}\cdot wA_{ij})}{MFC_{i}}, & e_{ij}\in E,\\ 0, & otherwise, \end{array}\right.$$

where

- $FA_{ij}$ is called Friendship Action, and may be a Comment, a Like, or any other action from user *i* on user *j* on a social network;
- $wA_{ij}$ is the weight of the Action $FA_{ij}$ and represents its importance over other actions. Its value is between 0 and 1. For example, for Facebook, a Comment is considered more important than a Like in the proposal. In particular, in the proposal, the weight of a positive Comment is 0.727, while the weight of a Like is 0.273, according to the values obtained from the data shown in the survey [20]. The system may detect positive, negative and neutral Comments using machine learning algorithms [21];
- $MFC_{i}$ is called the Maximal Friendship Coefficient, and represents the maximum sum of weighted actions performed by a user *i*. Its value is: $max_{j}\left(\sum \left(FA_{ij}\cdot wA_{ij}\right)\right)$.

Thus, the $df_{ij}$ value is considered the degree of friendship from user *i* to user *j*.

The remaining scores are obtained from the assessments of users after sharing cars. When a route is completed, the users who participated in it can vote between 1 and 5 stars. Each passenger individually assesses the driver, and the driver individually assesses each passenger.

The methodology used for determining the rating estimation for drivers and passengers is based on the risk assumed by each one. This risk has been established taking into account the number of verifiable data, the elements that can be damaged, and the greater importance attached to the supply rather than the demand. With this in mind, it is assumed that a driver has more verifiable data and can suffer more damage as he/she provides the vehicle, with all its features and an up-to-date insurance. Thus, at least initially, the system assigns greater value to users who offer empty seats in their cars than to passengers who are looking for empty seats because, without an offer, it is impossible to meet a demand. Therefore, the scores of Table 2 have been adjusted according to that argument and taking into account that a parameter greater than three stars is considered a positive rating, less than three is negative, and equal to three is neutral. In addition, the system gives the driver added impact due to the fact that he/she provides more verifiable information.

The final metric rating of the assessments given to a user *i* by other users in the proposal is computed through $ar_{i}$, the average of all the ratings received by *i* measured in points. A user who has been using the system for a while normally would have more ratings than a recent user, unless the behaviour of the first user has been incorrect. Thus, the way the proposed system can compare the performance of both users is by averaging their ratings because, otherwise, if the simple sum of ratings were used, the user who has been using the system longer would always have more points than a recent one, although the recent one has had a better behaviour or performance.

Finally, the metric $TR_{ij}$ (Trust Rate) indicating the degree of trust in user *i* from user *j*, is given by Equation (2):

(2) $$TR_{ij}=\left\{\begin{array}{cc} DT_{ij}, & \mathrm{if}\;\mathrm{users}\;i\;\mathrm{and}\;j\;\mathrm{are}\;\mathrm{direct}\;\mathrm{friends}\;,\\ IT_{ij}, & \mathrm{if}\;\mathrm{some}\;\mathrm{friendship}\;\mathrm{chain}\;\mathrm{exists}\;\mathrm{from}\;\mathrm{user}\;i\;\mathrm{to}\;\mathrm{user}\;j,\\ NT_{ij}, & \mathrm{if}\;\mathrm{no}\;\mathrm{friendship}\;\mathrm{chain}\;\mathrm{exists}\;\mathrm{from}\;\mathrm{user}\;i\;\mathrm{to}\;\mathrm{user}\;j, \end{array}\right.$$

where

- If users *i* and *j* are direct friends, Direct Trust (DT) is obtained using Equation (3)
  (3) $$DT_{ij}=df_{ij}\cdot wF+ar_{i}\cdot wR.$$
- If some chain of friends from user *i* to user *j* exists, Indirect Trust (IT) is given by Equation (4)
  (4) $$IT_{ij}=F\left(i\to j\right)\cdot wF+ar_{i}\cdot wR.$$
- If there is no friendship chain from user *i* to user *j*, Equation (5) is applied
  (5) $$NT_{ij}=ar_{i}\cdot wR,$$

where

- wF is called weight of Friendship, and its value (0.625) is obtained by the average happiness of people with their friends in the social network of Facebook [22];
- wR is the weight of Rating, whose value is obtained using 1−wF, so it is 0.375;
- F(i→j) gives the degree of friendship from user *i* to user *j* obtained through the friendship chain $\left\{i,i_{1},i_{2},....,i_{c},j\right\}$ between both. This value is obtained considering the probability of independent events. $F\left(i\to j\right)=df_{ii_{1}}\cdot df_{i_{1}i_{2}}\cdot \cdot \cdot df_{i_{c}j}$. This value is always between 0 and 1, and decreases along with the length of the friendship chain.

The relationship between the metric of the trust rate $TR_{ij}$ and the character shown to the user *j* is shown in Table 3.

The maximum trust rate that a user can get is 1 (represented as *A* score), which corresponds to the situation when the user direct friends have rated him/her with the highest scores. On the other hand, a user has *F* valuation if he/she does not have any degree of friendship or is starting to be known, and/or has had mostly negative reviews.

This valuation is dynamically calculated as a function of the friendship degree that a user has in each moment, and the ratings he/she has received by his/her use of the platform till then. Thus, the system helps users to have an up-to-date reliability measure about whether to trust another user or not. In addition, only users who have a valuation higher than *B* and/or users who have been accepted by the driver to make the route can see certain driver data, such as the phone number or other specific data. The system threshold for reliability has been set to a value higher than *C*, that is *A* or *B*, because this is the minimum value that represents direct friendship.

The reputation algorithm to compute trust rates is run in parallel using different threads to optimize efficiency. For the calculation of IT value, where two users are not direct friends, but they share a common chain of friendship, and each partial degree of friendship between direct friends is calculated in a different thread.

In order to discern whether an action taken on social network is positive or negative, the scheme uses AlchemyAPI based on Machine Learning algorithms to provide the social sentiment of a text. In addition, the proposal only takes into account the latest 100 actions of interactions between users on social networks. Thus, each $TR_{ij}$ value is computed dynamically for each pair of users *i* and *j* as it depends on the degree of friendship between the pair of users and their last interactions in social networks.

**Figure 2:**
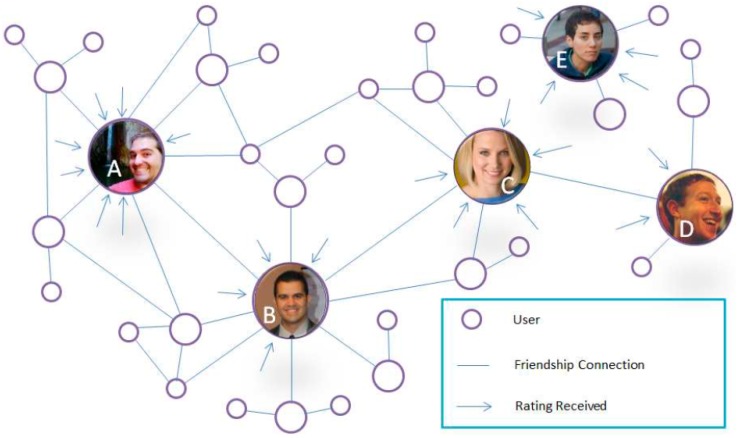
Example of Graph Representing Friendship in a Social Network.

In order to show a didactic example of the operation of the proposed reputation algorithm, the network shown in Figure 2 is considered to generate the Degrees of Friendship included in Table 4.

Table 5 shows the Ratings received by the users within the application after completed routes.

Thanks to the data in Table 4 and Table 5, the Trust Rates between each pair of users shown in Table 6 have been computed.

For example, in order to compute the trust rate $TR_{AB}$ in user A regarding his relationship with user B, the required calculations are as shown in Equation (6).

(6) $$\begin{array}{c} DT_{AB}=\frac{\left(78\times 0.273)+(19\times 0.727\right)}{40.822}\times 0.625+\frac{0.75+1+0.5+0.5+0.75+0.5}{6}\times 0.375\;\\ =\frac{35.107}{40.822}\times 0.625+\frac{4}{6}\times 0.375\;\\ =0.537+0.25=0.787. \end{array}$$

To compute the trust rate $TR_{DA}$ in user D regarding his relationship with user A, the required calculations are as shown in Equation (7)

(7) $$\begin{array}{c} IT_{DA}=\frac{\left(42\times 0.273)+(7\times 0.727\right)}{40.876}\times \frac{\left(4\times 0.273)+(2\times 0.727\right)}{41.738}\times \frac{\left(82\times 0.273)+(12\times 0.727\right)}{40.880}\times 0.625\\ \;\;\;\;\;\;\;\;\;\;\;\;\;\;\;+\frac{0.15+0.25+0.15}{3}\times 0.375\\ \;\;\;\;\;\;\;\;\;\;=\left(0.405\times 0.061\times 0.761\right)\times 0.625+\frac{0.55}{3}\times 0.375\\ \;\;\;\;\;\;\;\;\;\;=0.019\times 0.625+0.183\times 0.375\\ \;\;\;\;\;\;\;\;\;\;=0.012+0.069=0.081. \end{array}$$

**Figure 3:**
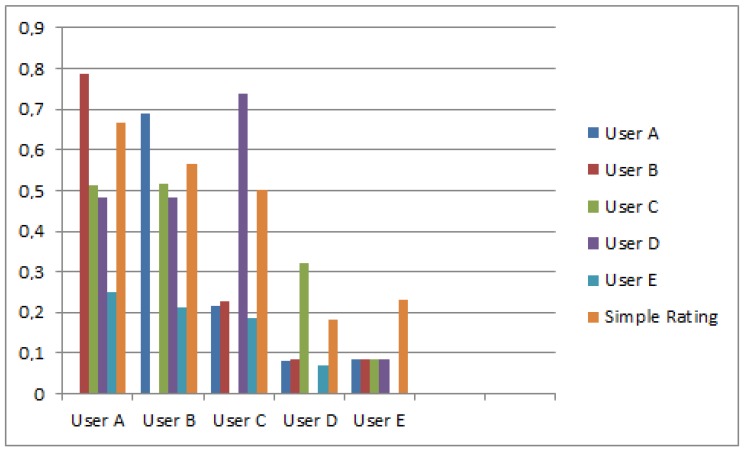
Comparison with Simple Rating-Based Systems.

The proposed system is fully dynamic and depends on the relationships between each pair of users. Figure 3 shows a comparison between the trust rates of each pair of users and the classical simple rating used in other proposals that are based only on ratings received from users.

## 5. The Android Application

**Figure 4:**
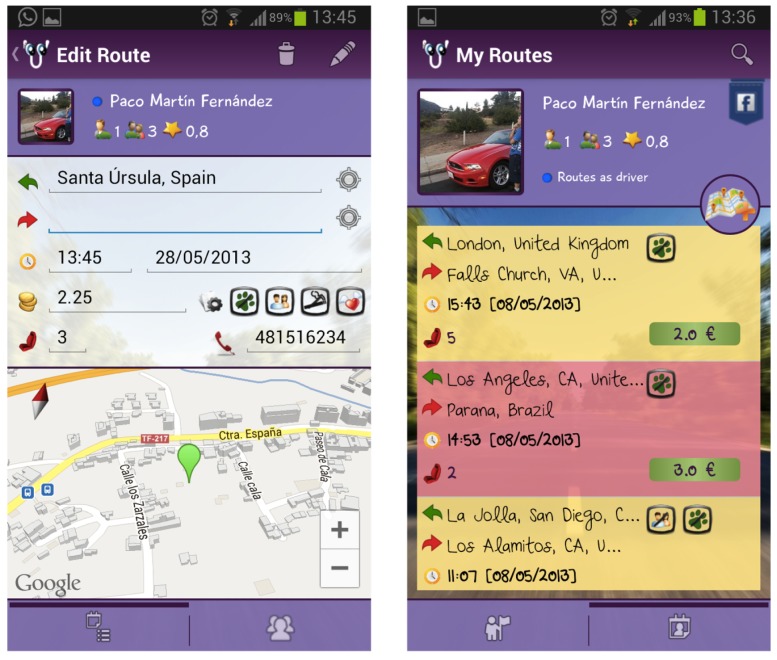
Carpoolap Screens: Route Edition and Routes List.

The proposed design has been embodied in an Android application that is already published in the Google Play Store under the name ’Carpoolap’ (see Figure 4).

The Android application is developed for the versions 3.0 or higher of the operating system. APIs like Google Maps v3.0, Google Places, Google Cloud Messaging, etc., and Facebook SDKs 3.0 and libraries like Action Bar Sherlock are used for the functionality of the new versions of Android on older versions. Autocomplete in address searches, Google Maps 3D Technology, design based on the latest versions of Android, push notifications with requests or responses of passengers or drivers, etc. are among the features of the Android Application.

**Figure 5:**
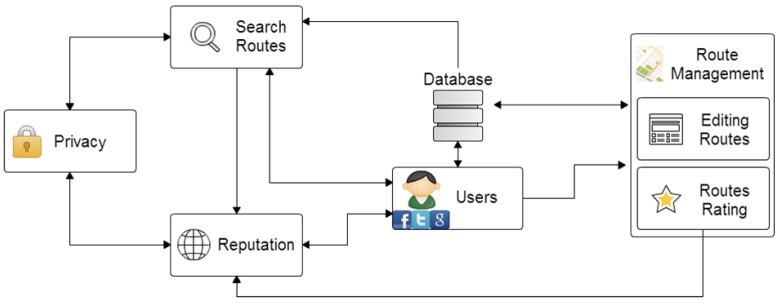
Use Model of the Implemented Application.

The use model of the implemented application is shown in Figure 5. Each user can see the routes he/she proposes as driver, and whether potential passengers exist for those routes. In addition, with colour codes, he/she can know the routes that each user has already made and the routes that have been confirmed by users. For the assessment of users participating in a route, after finishing it, each one can give a score. In order to deploy the carpooling platform, a server was developed using JavaScript technologies by frameworks like ‘node.js’ and ‘express.js’. As a database for all the centralized data on this server, a No SQL database, such as MongoDB [23], was adopted. The server was deployed on a micro instance of Amazon Web Services, specifically under an Ubuntu machine with Amazon EC2 account.

Although the Carpoolap application that includes the proposed trust algorithm has been published in the Google Play Store, the lack of marketing campaign and consequent lack of users means that data from the real application are not enough to extract useful conclusions.

## 6. Security Analysis of the Scheme

Regarding the security of the platform, Sybil attack is the most notorious attack in traditional carpooling systems. This type of attack is a hacking attack on peer-to-peer networks where a malicious device illegitimately takes multiple identities by forging them. Due to the privacy-preserving environment of carpooling schemes, Sybil vulnerability is generally hard to defend against.

In a Sybil attack, the attacker subverts the reputation system of a peer-to-peer network by creating a large number of pseudonymous identities, using them to gain a disproportionately large influence. The vulnerability of a reputation system to a Sybil attack depends on how cheaply identities can be generated, the degree to which the reputation system accepts inputs from entities that do not have a chain of trust linking them to a trusted entity, and whether the reputation system deals with all entities in the same way.

An entity of the analysed peer-to-peer network is a piece of software that has access to local resources. It advertises itself on the peer-to-peer network by presenting an identity. However, more than one identity could correspond to a single entity. In other words, the mapping of identities to entities could be many to one. Entities in peer-to-peer networks could use multiple identities for purposes of redundancy, resource sharing, reliability and integrity because, in peer-to-peer networks, the identity is used as an abstraction so a remote entity could be aware of other identities without necessarily knowing the correspondence of identities to local entities. By default, each different identity is usually assumed to correspond to a different local entity. However, actually, many identities may correspond to the same local entity.

A dishonest member or an adversary node may present multiple identities to a peer-to-peer network in order to appear and work as multiple distinct nodes. After becoming part of the peer-to-peer network, the adversary may then overhear communications or act maliciously. By masquerading and presenting multiple identities, the adversary can control the network substantially.

The proposed reputation algorithm reduces significantly the vulnerability to the Sybil attack described above because most of the score of the reputation algorithm is preceded by confidence in the degrees of friendship that binds each user to another user. Thus, if a user does not know (at all) another user, very high ratings of the latter in the system are not enough for guaranteeing reliability for the first user because, in the proposed system, the weight of the average rating is 37.5% of the total trust rate, while the weight of the degree of friendship is 62.5%.

## 7. Conclusions

This paper proposes a new system to increase the safety and reliability of existing carpooling systems, in order to overcome the psychological barrier that slows down the use of many potential users of carpooling. In order to achieve this objective, the system includes, on the one hand, a strong social component that fosters trust among users, and, on the other hand, progressive access to user data based on the estimation of the confidence levels provided by the system. The design of the proposal has been tested for its implementation in a smartphone application that supports the Android platform, is shared as an Open Source Project, and has been published in the Google Play Store. This work is in progress, so there are still several open issues, such as the development of the application on other platforms, the creation of an API for use in third-party applications or the study of existing agent-based models to compare the performance of the proposed system with the implementation of such mechanisms. 

## Figures and Tables

**Table 1:** Carpooling platforms.

Platform	Social Network	Privacy	Points System	Phone Cert	Trust Algorithm
Amovens [2]	yes	yes	yes	no	no
Blablacar [3]	yes	yes	yes	yes	no
Compartir.org [4]	no	yes	no	no	no
Zimride [5]	yes	yes	no	no	no
**Our system**	**yes**	**yes**	**yes**	**no**	**yes**

**Table 2:** Impact of the ratings.

Star Rating	Driver Rating Impact	Passenger Rating Impact
1 star	0.15 points	0.00 points
2 stars	0.25 point	0.15 points
3 stars	0.50 points	0.25 points
4 stars	0.75 points	0.50 points
5 stars	1.00 points	0.75 points

**Table 3:** Equivalence between Trust Rate and Character Shown to User.

Trust Rate	Character Shown to User
[0–0.15)	*F*
[0.15–0.25)	*E*
[0.25–0.5)	*C*
[0.5–0.75)	*B*
[0.75–1]	*A*

**Table 4:** Example of Degrees of Friendship.

Relationship	Data about Relationship	
User A–User B	Likes in Facebook	78
	positive Comments in Facebook	19
	*degree of friendship*	*0.860*
User B–User A	Likes in Facebook	82
	positive Comments in Facebook	12
	*degree of friendship*	*0.761*
User B–User C	Likes in Facebook	53
	positive Comments in Facebook	8
	*degree of friendship*	*0.497*
User C–User B	Likes in Facebook	4
	positive Comments in Facebook	2
	*degree of friendship*	*0.061*
User C–User D	Likes in Facebook	76
	positive Comments in Facebook	21
	*degree of friendship*	*0.882*
User D–User C	Likes in Facebook	42
	positive Comments in Facebook	7
	*degree of friendship*	*0.405*

**Table 5:** Example of Received Ratings.

	User A	User B	User C	User D	User E
Received Ratings as Driver	4 stars 5 stars	3 stars 4 stars 4 stars	5 stars	-	2 stars 3 stars
Received Ratings as Passenger	4 stars 4 stars 5 stars 4 stars	5 stars 4 stars 2 stars	3 stars 4 stars 3 stars	2 stars 3 stars 2 stars	3 stars 1 star 2 stars

**Table 6:** Example of Trust Rates.

	User A	User B	User C	User D	User E
User A	-	0.787 (*A* Score)	0.512 (*B* Score)	0.480 (*C* Score)	0.250 (*C* Score)
User B	0.688 (*B* Score)	-	0.517 (*B* Score)	0.481 (*C* Score)	0.212 (*E* Score)
User C	0.216 (*E* Score)	0.226 (*E* Score)	-	0.739 (*B* Score)	0.187 (*E* Score)
User D	0.080 (*F* Score)	0.084 (*F* Score	0.322 (*C* Score)	-	0.069 (*F* Score)
User E	0.086 (*F* Score)	0.086 (*F* Score)	0.086 (*F* Score)	0.086 (*F* Score)	-
